# Epidemiology and clonality of carbapenem-resistant *Acinetobacter baumannii* from an intensive care unit in Palermo, Italy

**DOI:** 10.1186/1756-0500-5-365

**Published:** 2012-07-20

**Authors:** Caterina Mammina, Daniela Maria Palma, Celestino Bonura, Aurora Aleo, Teresa Fasciana, Concetta Sodano, Maria Antonietta Saporito, Maria Stella Verde, Cinzia Calà, Andrea Neville Cracchiolo, Romano Tetamo

**Affiliations:** 1 Department of Sciences for Health Promotion “G. D’Alessandro”, Section of Hygiene, University of Palermo, Palermo, Italy; 2 II Intensive Care Unit, ARNAS General Hospital “Civico & Benfratelli, Palermo, Italy; 3 Department of Sciences for Health Promotion “G. D’Alessandro”, Section of Microbiology, University of Palermo, Palermo, Italy; 4 Laboratory of Microbiology, ARNAS General Hospital “Civico & Benfratelli, Palermo, Italy

## Abstract

**Background:**

Multidrug-resistant *Acinetobacter baumannii*, initially considered as having a poor clinical relevance, is frequently isolated from infection cases in intensive care units. We describe the epidemiology of carbapenem resistant *A. baumannii* (CRAB) in a general ICU in Palermo, Italy, from October 2010 to March 2011.

**Findings:**

58 of 61 isolates exhibited MICs for meropenem or imipenem ≥16 mg/L. Forty-nine carried *bla*_OXA-23_ and two *bla*_OXA-58_ genes.

Five subtype clusters were detected by rep-PCR. Clusters D and E included 10 isolates that tested negative for the carbapenem resistance genes. MLST attributed all isolates, but two, with sequence type (ST)2, whereas the two remaining isolates with ST78.

The respiratory tract was the most common site of infection (26 out of 36 cases. 72.2%). A high infection related mortality rate was observed (18 out of 35 patients, 51.4%). Nineteen patients tested positive for other multidrug resistant organisms in addition to CRAB. In eight cases isolates belonging to distinct subtype clusters and/or with distinct carbapenemase profiles were identified.

**Conclusions:**

Carbapenem resistance was prominently driven by the dissemination of CRAB isolates belonging to ST2, carrying the carbapenemase gene *bla*_OXA-23_. The colonization/infection of some patients by multiple strains is suggestive of an endemic circulation of CRAB.

## Findings

Eighty-four consecutive CRAB isolates were recovered from 36 patients, who were admitted to the ICU under study between October 1, 2010 and March 31, 2011.

Based upon the inclusion criteria, 61 isolates were submitted to testing by PCR analysis to confirm the presence of carbapenemase genetic determinants and molecular typing by rep-PCR and, for representative isolates, by MLST. A number of isolates from each patient ranging between one and six was analyzed. The clinical samples more prevalent were bronchial aspirates (n. 29), followed by swabs from wound or tracheostomy (n. 8), blood (n. 7), drainage fluids and tips of central venous catheter (n. 6 each).

Among the 61 isolates, all (100%) were resistant to penicillins (ampicillin, carbenicillin), cephalosporins (cefepime, cefotaxime, ceftazidime), β-lactam-β-lactamase inhibitor combinations and fluoroquinolones, whereas susceptibility to aminoglycosides [amikacin, four (6.5%) susceptible and two (3.3%) intermediate; gentamicin, one (1.6%) susceptible and nine (14.7%) intermediate; tobramicin, six (9.8%) susceptible and 12 (19.7%) intermediate) and sulfamethoxazole-trimethoprim, nine (14.7%) susceptible] was variable. Moreover, 50 (82.0%) and 11 isolates (18.0%) tested, respectively, susceptible (MICs 0.5 – 2 mg/L) and intermediate (MIC 4 mg/L) to tigecycline. Only colistin showed 100% susceptibility with MICs ranging between 0.5 and 2 mg/L. Three strains only had a MIC for imipenem of 8 mg/L, whilst all the remaining isolates exhibited MICs ≥ 16 mg/L. Moreover, MICs for meropenem were ≥ 16 mg/L for all, but three strains that showed MICs of 4 and 8 mg/L in one and two cases, respectively.

The 61 CRAB strains were investigated for the presence of carbapenemase genes. All isolates harboured a *bla*_OXA-51-like_ sequence. Forty-nine isolates had, in addition, *bla*_OXA-23_ and two only *bla*_OXA-58_ genes. No MBL (IMP and VIM) gene sequence was detected. The two OXA-58 isolates had MICs for imipenem ≥ 16 mg/L, whereas for meropenem of 4 and 8 mg/L, respectively. Ten isolates for which the imipenem MIC was ≥ 8 mg/L had an unidentified carbapenem resistance mechanism. All tested negative for the *bla*_OXA-143_ sequence. Presence of IS*Aba*1 in the promoter region of the *bla*_OXA-51-like_ gene was not investigated because of the inconsistent literature findings about its correlation with carbapenem resistance and the possible involvement of alternative mechanisms [1,2].

**Figure 1:**
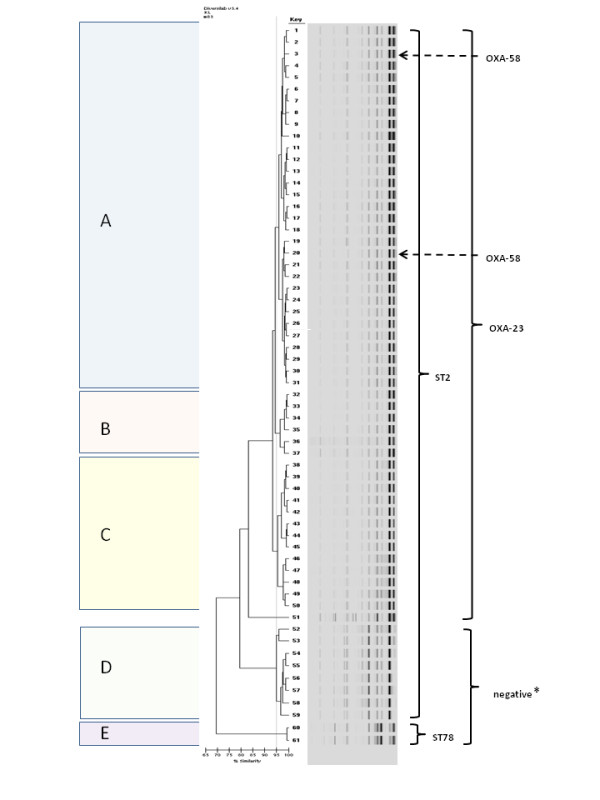
**Dendrogram and computer-generated image of rep-PCR banding patterns of the 61 CRAB isolates under study.** Similarity calculation is based upon the Kullback–Leibler method, clustering is based upon the Unweighted Pair Group Method with Arithmetic Mean (UPGMA)

By adopting a similarity coefficient of ≥95% as the threshold, all isolates, but one, clustered into five distinct groups named A to E (Fig.1). Thirty-one out of 61 clustered in a large group that included 29 *bla*_OXA-23_ and the two *bla*_OXA58_ isolates. Two further subtype clusters, B and C, included 6 and 13 OXA-23 producing isolates, respectively. Strains belonging to the subtype clusters A, B and C clustered at a 94% similarity level. Cluster D and cluster E grouped eight and two isolates, respectively, that tested negative for the carbapenem resistance genes under investigation. One isolate only did not cluster.

MLST attributed all isolates, but two, with sequence type (ST)2, whereas the two remaining isolates belonged to ST78.

There was not apparent relationship between subtype clusters, carbapenem resistance genes and the whole resistance patterns or carbapenem resistance phenotype. Twelve out of 14 isolates showing susceptibility or intermediate susceptibility to at least one aminoglycoside were isolated in the first three months of the investigation.

The main demographic and clinic characteristics of the 36 patients are summarized in Table 1. The respiratory tract was the most common site of infection (26 out of 36 cases, 72.2%). Moreover, a high infection related mortality rate was observed (18 out of 35 patients with a known outcome, 51.4%). From 19 patients other MDR organisms in addition to CRAB were isolated from at least one clinical sample (Table 1). Of special interest, eight patients were co-colonized or co-infected at the same site of CRAB by *Klebsiella pneumoniae* carbapenemase-producing *Klebsiella pneumoniae* (KPC-Kp).

**Table 1:** Demographic and clinical characteristics of the patients admitted in the period October 1, 2010 – March 31, 2011 and infected by CRAB

**patient**	**age/gender**	**reason for admission**	**underlying disease**	**SAPSII**	**type of infection**	**other MDR organism(s) isolated**	**clinical picture**	**outcome**
1	77/M	thoracic trauma	hypertension	39	cholecystitis	none	severe sepsis	discharge
2	78/F	respiratory failure	lung cancer	31	LRTI	none	sepsis	death
3	22/F	polytrauma	none	27	RTI	none	sepsis	discharge
4	20/M	cranial trauma	none	42	pneumonia	KPC-Kp	severe sepsis	death
5	64/M	AMI	hypertension	54	pneumonia	KPC-Kp	septic shock	death
6	32/F	respiratory failure	major haematological disease	30	pneumonia	none	septic shock	death
7	34/F	polytrauma	none	45	LRTI	*P. aeruginosa*	sepsis	discharge
8	19/M	polytrauma	none	48	pneumonia	MRSA, *P. aeruginosa*	severe sepsis	discharge
9	73/M	acute abdomen	hypertension, diabetes	38	peritonitis	KPC-Kp	septic shock	death
10	50/M	polytrauma	none	44	pneumonia	none	severe sepsis	discharge
11	74/F	respiratory failure	diabetes, haematological malignancy	92	pneumonia	*P. aeruginosa*	septic shock	death
12	73/F	post-surgical admission	hypertension, diabetes, COPD	18	complicated SSTI	KPC-Kp	sepsis	discharge
13	64/M	severe metabolic imbalance	diabetes	71	pneumonia	*P. aeruginosa*	septic shock	death
14	39/F	respiratory failure	autoimmune disease, HCV	37	pneumonia	ESBL-Kp	septic shock	death
15	60/M	polytrauma	hypertension	26	pancreatitis	KPC-Kp	septic shock	death
16	60/M.	third degree burns	hypertension	71	complicated SSTI	none	septic shock	death
17	70/M	encephalitis	hypertension	50	LRTI	KPC-Kp	severe sepsis	death
18	75/F	acute abdomen	hypertension, diabetes	32	peritonitis	none	u.i.	u.i.
19	35/F	polytrauma	none	31	LRTI	MRSA	sepsis	discharge
20	71/M	stroke	hypertension, cerebral vasculopathy	48	pneumonia	none	severe sepsis	discharge
21	69/M	AMI	hypertension, arrhythmia	56	pneumonia	none	septic shock	death
22	60/M	metabolic coma	hepatopathy	71	LRTI	none	severe sepsis	death
23	76/F	respiratory failure	COPD, diabetes	48	pneumonia	none	septic shock	death
24	64/M	post-surgical admission	basalioma	21	complicated SSTI	KPC-Kp, ESBL-Ec	cardiac arrest	death
25	68/M	post-surgical admission	autoimmune disease, COPD, arrhythmia	43	pneumonia	none	severe sepsis	discharge
26	23/M	polytrauma	none	u.i.	BSI	none	septic shock	death
27	18/M	polytrauma	none	30	LRTI	KPC-Kp	sepsis	discharge
28	20/F	respiratory failure	SLE	u.i.	cystitis	MRSE	sepsis	discharge
29	16/M	cranial trauma	epilepsy	u.i.	sinusitis	none	sepsis	discharge
30	70/M	ruptured aneurism	hypertension, COPD, diabetes	23	LRTI	*P. aeruginosa*	severe sepsis	discharge
31	28/M	polytrauma	none	17	LRTI	*P. aeruginosa*	sepsis	discharge
32	75/M	respiratory failure	hypertension, diabetes, CKD	27	pneumonia	none	septic shock	death
33	58/M	respiratory failure	hypertension, COPD, hearth failure	34	pneumonia	none	severe sepsis	discharge
34	41/M	polytrauma	none	38	LRTI	MRSA	severe sepsis	discharge
35	87/F	acute abdomen	coagulopathy	45	pneumonia	none	severe sepsis	discharge
35	64/M	metabolic coma	diabetes	71	pneumonia	*P aeruginosa*	septic shock	death

AMI = acute myocardial infarction; BSI = bloodstream infection; CKD = chronic kidney disease; COPD = chronic obstructive pulmonary disease; LRTI = lower respiratory tract infection; RTI = reproductive tract infection; SLE = Systemic Lupus Erythematosus; SSTI = skin and soft tissue infection; KPC-Kp = KPC carbapenemase-producing *Klebsiella pneumoniae*; ESBL-Kp = Extended spectrum β-lactamases producing *Klebsiella pneumoniae*; ESBL-Ec = Extended spectrum β-lactamases producing *Escherichia coli*; MRSA = methicillin resistant *Staphylococcus aureus*; MRSE = methicillin resistant *Staphylococcus epidermidis*; SAPSII = Mean Simplified Acute Physiology Score II; u.i = unavailable information.

From 19 out of 36 patients two to six CRAB isolates were consecutively identified in the period under study. In 11 patients an indistinguishable CRAB isolate was repeatedly identified, unlike from the remaining eight patients. In particular, three out of these patients yielded isolates belonging to distinct subtype clusters and/or with distinct carbapenemase profiles in different clinical samples obtained up to 24 hours apart from each other; alternatively, distinct isolates were sequentially identified from the same or different clinical samples from five further patients within intervals of time ranging between four and seventy days (Table 2).

**Table 2:** Source, time of isolation, carbapenem MICs and molecular characteristics of the replicate CRAB isolates from the eight patients yielding distinct isolates by subtype cluster and/or carbapenemase profile

patient*	**isolate**	**date of isolation**	**clinical sample**	**MIC imipenem**	**MIC meropenem**	**PCR**	**rep-PCR cluster**	**ST**
3	33	October 13, 2010	BA	> = 16	> = 16	OXA-23	B	2
	18	October 14, 2010	CVC	> = 16	> = 16	OXA-23	A	2
4	51	October 25, 2010	BA	> = 16	> = 16	OXA-23	singleton	2
	29	December 1, 2010	CSF	> = 16	> = 16	OXA-23	A	2
	46	December 28, 2010	CSF	> = 16	> = 16	OXA-23	C	2
9	19	November 22, 2010	peritoneal fluid	> = 16	> = 16	OXA-23	A	2
	32	November 22, 2010	BA	> = 16	> = 16	OXA-23	B	2
	53	November 23, 2010	blood	> = 16	> = 16	negative	D	2
15	13	December 27, 2010	BA	> = 16	> = 16	OXA-23	A	2
	17	January 7, 2011	drainage fluid	> = 16	> = 16	OXA-23	A	2
	30	January 8, 2011	blood	> = 16	> = 16	OXA-23	A	2
	16	January 19, 2011	drainage fluid	> = 16	> = 16	OXA-23	A	2
	14	January 29, 2011	wound swab	> = 16	> = 16	OXA-23	A	2
	61	February 14, 2011	blood	8	> = 16	negative	E	78
17	44	January 3, 2011	BA	> = 16	> = 16	OXA-23	C	2
	11	January 7, 2011	blood	> = 16	> = 16	OXA-23	A	2
23	48	January 24, 2011	drainage fluid	8	8	OXA-23	C	2
	6	January 25, 2011	tracheostomy swab	> = 16	> = 16	OXA-23	A	2
24	60	January 24, 2011	BA	> = 16	> = 16	negative	E	78
.	45	April 4, 2011	wound swab	> = 16	> = 16	OXA-23	A	2
27	41	February 21, 2011	BA	> = 16	> = 16	OXA-23	C	2
	23	March 11, 2011	tracheostomy swab	> = 16	> = 16	OXA-23	A	2

* patient’s numeric code refers to Table 1.

BA, bronchial aspirate; CVC, central venous catheter; CSF, cerebrospinal fluid.

## Background

The emergence of multidrug-resistant *Acinetobacter baumannii* strains is posing severe challenges in many clinical and post-acute care settings [3,4]. Initially considered as a colonizing bacterial species with poor clinical relevance, it is being isolated more and more frequently, especially in the intensive care units (ICUs), as the etiological agent of serious infections, such as ventilator-associated pneumonia (VAP), infections of bloodstream, urinary tract, central nervous system and wounds [5]. *A. baumannii* is typically selected by prior antimicrobial treatments, but in turn infections caused by this organism are difficult to treat, so leading to increasingly rely on last resort molecules, such as polymyxins and tigecycline for therapy [6]. Additionally, infections with carbapenem-resistant *A. baumannii* (CRAB) organisms may require discharged patients from acute hospitals to be further managed in long-term care facilities (LTCFs) or alternative post-acute care facilities and impose diagnostic, therapeutic and infection-control extra-costs [7,8].

Isolates of *A. baumannii* carry a naturally occurring *bla*_OXA-51_ β-lactamase with a weak carbapenemase activity. Moreover, in a great proportion of isolates from different geographical areas, carbapenem resistance in *A. baumannii* is mediated by the acquisition of a class B or a class D carbapenem-hydrolyzing enzymes [9]. In Italy, *bla*_OXA-58_ has been reported to be the most prevalent OXA type carbapenem resistance gene, but a shift towards *bla*_OXA-23_ that appears to be gradually substituting *bla*_OXA-58_ has been recently described [10-12]. This trend is likely to be related to the a higher carbapenemase activity of *bla*_OXA-23_ than *bla*_OXA-58_ that results in higher carbapenem MICs and a consequent selective advantage. However, large scale data on the prevalence of these genes throughout Italy are still scarce.

Genotypic characterization of *A. baumannii* has shown that distinct clonal lineages, the so-called European (EU) clones I, II and III, are widely spread across Europe and include strains that are usually multiresistant (MDR) and responsible for epidemic and endemic occurrence of healthcare associated colonization and infection [3].

The objective of our study was to analyze the spread and clonality of CRAB in a general ICU in Palermo, Italy, by prospectively collecting and characterizing isolates from infected patients who were being admitted to the ICU over a six-month period from October 2010 to March 2011. Medical records of patients from whom CRAB was isolated were also retrospectively reviewed to investigate their clinical outcomes.

## Methods

### Setting

The 2^nd^ ICU of the ARNAS “Civico & Benfratelli” General Hospital of Palermo, Italy, is a 10-bed medical-surgical Unit with approximately 430 admissions per year. Preexisting medical and surgical conditions are generally present in approximately 45% and 55%, respectively, of all admissions. Organ failure is the leading cause of admission (70%), followed by monitoring/weaning from mechanical ventilation (30%). ICU mortality is about 25%. Nurse to patient ratio is 1:2.

Consecutive CRAB isolates were recovered from all patients who were admitted to 2^nd^ Intensive ICU during the six-month period October 1, 2010 – March 31, 2011. For the purpose of this study, the following inclusion criteria were used for the selection of CRAB isolates to be submitted to molecular typing: a) all the unique isolates from different patients; b) replicate isolates obtained from different clinical samples of the same patient up to 24 hours apart from each other; c) replicate isolates obtained from the same patient more than 24 hours apart from each other from whatever clinical sample.

CRAB isolates were isolated by standard methods and identified by the Vitek 2 automated microbiology system (bioMérieux, Marcy l'Étoile, France). Antimicrobial susceptibility testing was automatically undertaken by using the commercial microdilution method Vitek 2. The MICs of imipenem and meropenem were determined by using the epsilometer test (Etest, AB Biodisk, Solna, Sweden). Results were interpreted according to Clinical and Laboratory Standards Institute (CLSI) criteria for *Acinetobacter* spp [13]. Due to the lack of standardization for *A. baumanniiEnterobacteriaceae* breakpoints (≤2 μg/ml, susceptible; ≥8 μg/ml, resistant) were used for interpreting the results of the tigecycline assays. The control strain used was *Escherichia coli* ATCC 25922.

### Characterization of CRAB isolates

To detect the presence of the most common carbapenemases, multiplex PCR was performed with primers that anneal to *bla*_OXA-51_*bla*_OXA-23_*bla*_OXA-24_ and *bla*_OXA-58_ carbapenemases and the MBLs *bla*_IMP_ and *bla*_VIM_, as previously described [14,15].

The newly described gene *bla*_OXA-143_ was also searched for [16].

To investigate the relationship among the CRAB isolates collected during the study period, we used the rep-PCR DiversiLab Microbial Typing System® (bioMérieux, Marcy l'Étoile, France), which amplifies the regions between the non-coding repetitive sequences in bacterial genomes. Extraction of DNA was performed using the UltraCleanTM Microbial DNA Isolation Kit (Mo Bio Laboratories, Inc., Carlsbad, CA, USA). Rep-PCR was performed using the DiversiLab *Acinetobacter* kit. DNA fragment separation and detection were done using the Agilent 2100 Bioanalyzer (Agilent Technologies, Santa Clara, CA, USA) and results were analyzed and interpreted using the Kullback–Leibler method, as previously reported [17]. Isolates were defined as genetically related when ≥95% similarity was identified.

Genotyping by multilocus sequence typing (MLST) was performed on representative CRAB strains selected on the basis of clustering by rep-PCR and carbapenemase gene pattern. MLST was based on the sequence analysis of the internal portions of seven housekeeping genes (*cpn*60, *fus*A, *glt*A, *pyr*G, *rec*A, *rpl*B and *rpo*B). All PCR amplifications were carried out under the following conditions: 30 cycles (denaturation at 94 °C for 1 min, annealing at 53 °C for 1 min, and extension at 72 °C for 1 min) preceded by a 5-min denaturation at 94 °C and followed by a 10-min extension at 72 °C. PCR products were sequenced in both directions by using BigDye fluorescent terminators and primers on a 310 DNA analyzer (Applied Biosystems, Warrington, United Kingdom). Details of the MLST scheme including amplification and sequencing primers, allele sequences and STs are available at Institute Pasteur's MLST Web site (http://www.pasteur.fr/mlst).

### Patients characteristics

The medical records of patients from whom CRAB was isolated were retrospectively reviewed. The following demographic and clinical data were obtained: age, gender, reason for admission, underlying diseases, type of infection and clinical manifestation, Mean Simplified Acute Physiology Score II (SAPS II), isolation of other MDR organisms, such as ESBL producing enterobacteria, vancomycin-resistant *Enterococcus* spp. (VRE) and methicillin-resistant *Staphylococcus aureus* (MRSA), if any, and outcome (dead/discharged).

This research conformed to local legislation and the Helsinki Declaration and was approved by the ethics committee of the ARNAS General Hospital “Civico & Benfratelli, Palermo, Italy.

## Discussion

CRAB is an healthcare serious issue in many European countries. Literature shows that carbapenem resistance rates are higher in southern Europe countries, such as Turkey, Greece, Spain and Italy [18]. In Europe, outbreaks are especially attributable to two main *A. baumannii* clones (the so-called European clones I and II) [19]. Clone II has been repeatedly reported in many European countries, including Italy [5,8-10]. With regard to the mechanism of carbapenem resistance in the Italian CRAB isolates, the spread of carbapenemases belonging to the molecular class D OXA enzymes has been well documented [8-10,19-22].

Our study reveals that carbapenem resistance in the ICU under study was prominently driven by the dissemination of CRAB isolates belonging to ST2, the European clone II, carrying the carbapenemase gene *bla*_OXA-23_. Of interest, in comparison with the data obtained by previous studies conducted in Italy, as well also in other Mediterranean countries, the prevalence of *bla*_OXA-58_ carrying isolates appears to be drastically lower [10,21,22]. Emergence of an epidemic lineage carrying *bla*_OXA-23_ carbapenemase genes that is displacing a genetically closely related CRAB clone encoding OXA-58 has been previously described in central Italy in a study involving 10 ICU [8]. The higher level resistance to carbapenems conferred by OXA-23 is thought to be the success key of OXA-23 carrying epidemic strains under the selective pressure due to the increasing use of carbapenems, mainly in combination with fluoroquinolones, forced in turn by the epidemic spread of extended spectrum β-lactamase producing organisms [23].

Five subtype clusters were recognized by rep-PCR typing, with the first three – A to C – appearing to be closely related and all belonging to ST2, so suggesting the dissemination in the ICU of at least three well differentiated clones. Isolates belonging to two of them, D and E, tested negative for carbapenemase genes under study, except for *bla*_OXA-51-like_. The two isolates belonging to the subtype cluster E were attributed with ST78, the so called “Italian clone”. This clone, after being identified firstly in Naples, Italy, in 2006–2007 as an emerging epidemic clone, has been subsequently identified in other cities of southern Italy, Catania, and northern, Italy, Novara, accounting for a 13% proportion of isolates [10,20-22]. More recently, the same clone has been detected in ICUs, but also in home-care patients in Palermo, Italy [5]. Hence, ST78 seems to have largely disseminated in Italy [20-22].

The detection in a same patient of CRAB isolates belonging to different subtype clusters is a concerning finding. An underestimation of the event is very likely due to the routinely adopted microbiological diagnostic procedures, that are not aimed at detecting a mixed population of CRAB in patient or a clinical sample. Furthermore, because an active culture surveillance program was not in place in the ICU under study, prevalence of CRAB positive patients/sites is likely to be much higher and the relative proportion of the different strains misrepresented. Nevertheless, identification of distinct strains in an interval of time of 24 hours strongly suggests that some patients could be simultaneously or sequentially colonized or infected by multiple strains. The clinical and prognostic significance of such a finding deserves further investigation. The variety of subtype clusters could be likely interpreted as suggestive of a situation of endemicity where the selective advantage conferred by the high level carbapenem resistance supports the epidemic spread of the ST2 lineage carrying the *bla*_OXA-23_ determinant.

According with previous Authors, our data confirm that CRAB is one of the most challenging Gram-negative pathogens to control and treat in the ICUs, resulting in serious infections and substantial mortality [24,25]. Unfortunately, infection control measures used to contain the CRAB dissemination can be very difficult to be applied and substantially ineffective when it has become endemic. On the other hand, compelling evidence has accumulated about the contribution that contaminated surfaces make to the epidemic and endemic transmission of many MDR organisms and above all CRAB and MRSA [26,27].

## Conclusions

Spread in the healthcare settings of CRAB is posing extremely challenging questions about the most effective strategies to be adopted in the infected patient management as well as in the control and prevention of transmission within and between the healthcare facilities. A timely recognition and an accurate description of the CRAB strains and clones that are spreading in a defined area can contribute to obtain a more reliable epidemiological picture and to devise effective and targeted control measures.

## Abbreviations

CRAB, Carbapenem resistant A. baumannii; ICU, Intensive care unit; LTCF, Long term care facility; MDR, Multidrug resistant; MIC, Minimum inhibitory concentration; MLST, MultiLocus sequence typing; rep-PCR, Repetitive sequence polymerase chain reaction; ST, Sequence type; VAP, Ventilator-associated pneumonia.

## Competing interests

The authors declare that they have no competing interests.

## Authors' contributions

CM and DMP designed the study. CS, MAS and MSV were in charge of identify and collect strains and information about them. CB, AA,TF and CC carried out typing of isolates and characterization of resistance genetic determinants. DMP, ANC and RT provided the clinical data about patients. CM, DMP, CB and ANC analyzed the data and wrote the paper. RT supervised the study. All authors read and approved the final manuscript.
